# Influence of Agronomic Factors on Mycotoxin Contamination in Maize and Changes during a 10-Day Harvest-Till-Drying Simulation Period: A Different Perspective

**DOI:** 10.3390/toxins14090620

**Published:** 2022-09-05

**Authors:** Bernat Borràs-Vallverdú, Antonio J. Ramos, Carlos Cantero-Martínez, Sonia Marín, Vicente Sanchis, Jesús Fernández-Ortega

**Affiliations:** 1 AGROTECNIO-CERCA Center, Food Technology Department, University of Lleida, Rovira Roure 191, 25198 Lleida, Spain; 2 AGROTECNIO-CERCA Center, Department of Crop and Forest Sciences, University of Lleida, Rovira Roure 191, 25198 Lleida, Spain

**Keywords:** maize, deoxynivalenol, fumonisin, tillage system, nitrogen fertilisation, crop diversification, water activity, *Fusarium*, *Lumbricus terrestris*

## Abstract

Agronomic factors can affect mycotoxin contamination of maize, one of the most produced cereals. Maize is usually harvested at 18% moisture, but it is not microbiologically stable until it reaches 14% moisture at the drying plants. We studied how three agronomic factors (crop diversification, tillage system and nitrogen fertilization rate) can affect fungal and mycotoxin contamination (deoxynivalenol and fumonisins B_1_ and B_2_) in maize at harvest. In addition, changes in maize during a simulated harvest-till-drying period were studied. DON content at harvest was higher for maize under intensive tillage than using direct drilling (2695 and 474 μg kg^−1^, respectively). We found two reasons for this: (i) soil crusting in intensive tillage plots caused the formation of pools of water that created high air humidity conditions, favouring the development of DON-producing moulds; (ii) the population of *Lumbricus terrestris*, an earthworm that would indirectly minimize fungal infection and mycotoxin production on maize kernels, is reduced in intensive tillage plots. Therefore, direct drilling is a better approach than intensive tillage for both preventing DON contamination and preserving soil quality. Concerning the simulated harvest-till-drying period, DON significantly increased between storage days 0 and 5. Water activity dropped on the 4th day, below the threshold for DON production (around 0.91). From our perspective, this study constitutes a step forward towards understanding the relationships between agronomic factors and mycotoxin contamination in maize, and towards improving food safety.

## 1. Introduction

Maize is one of the most produced cereals worldwide and is used for both human consumption and animal feed. It is estimated that 1,162,352,997 tons of maize were produced in 2020 [1]. Unfortunately, maize is susceptible to toxigenic fungal contamination at all points of its supply chain (pre-harvest, harvest and post-harvest stages) [2,3]. Amongst the most prevalent and toxic fungal metabolites in maize, the mycotoxins fumonisin B_1_ (FB_1_), fumonisin B_2_ (FB_2_) and deoxynivalenol (DON) can be found [2,4]. In maize, fumonisins are primarily caused by *Fusarium verticillioides*, *Fusarium proliferatum* and *Fusarium subglutinans*, while DON is mostly caused by *Fusarium graminearum* and *Fusarium culmorum* [4,5,6,7]. Apart from being one of the major causes of economic losses in maize crops, mycotoxin contamination can have a severe impact on human and animal health.

FB_1_ affects sphingolipid metabolism, causes oxidative stress and can cause damage to cell DNA [8]. In humans, fumonisins have been associated with a higher risk of oesophageal carcinoma [9]. In animals, FB_1_ ingestion can cause leucoencephalomalacia (LEM) in horses, hepatocarcinogenesis in rats and pulmonary oedema in swine [10]. DON inhibits protein and DNA synthesis in eukaryotic cells, and can induce nausea, emesis, vomiting, skin inflammation, leukopenia, diarrhoea, haemorrhage in the lungs and brain, and the destruction of bone marrow [11,12]. The European Union (EU) regulates the maximum content of DON and the sum of FB_1_ + FB_2_ in certain foodstuffs (including maize) and provides guidance values for those and other mycotoxins in food and feed products [13,14,15].

Many factors can affect mycotoxin contamination in maize throughout the whole supply chain. Among them, we can find biological factors (susceptibility of the crop), environmental factors (temperature, rainfall, air relative humidity, insects/bird injuries), crop management (planting and harvest dates, tillage practices, fertilization, crop rotation, irrigation), crop harvesting (crop maturity, temperature, moisture, mechanical injury), transportation conditions, time until drying, and proper drying or storage conditions (aeration, temperature, pest/rodent control) [2,4,16].

The accepted commercial moisture for maize harvesting in NE Spain is around 18%. Sometimes, when the maize is almost ready for harvest, rain can increase the grain moisture, promoting mould proliferation and extending the period before harvesting until moisture reaches commercial standards again. In addition, in some areas drying facilities are undersized. Therefore, as all maize is harvested within an interval of a few days, it is usual to find huge amounts of maize grain outdoors waiting to be processed in the drying plants. This waiting period can sometimes be as long as 10 days. Despite the accepted commercial moisture for maize being about 18%, it has been reported that to ensure that no moulds can grow in grain nor produce mycotoxins, its maximum moisture content must be no more than 14% [17,18]. To our knowledge, there is no information about how this waiting period can influence fungal and mycotoxin contamination of the maize.

Hence, the objectives of our study were to: (i) study the impact of several agronomic factors (the crop diversification, the tillage system and the nitrogen (N) fertilization rate) on total fungal contamination, *Fusarium* spp. contamination and DON, FB_1_ and FB_2_ contaminations of recently harvested maize; (ii) simulate the waiting period between maize harvesting and drying for 10 days, and study the influence of waiting time and temperature on the previously mentioned variables.

## 2. Results and Discussion

### 2.1. Influence of Agronomic Factors on the Maize at Harvest Date

At harvest date (day 0) all the analyzed maize from both maturity groups, N fertilization rates and tillage systems was contaminated with DON (Table 1). On the other hand, only 12.5% of that same maize samples contained FB_1_ and FB_2_. Average concentrations of the contaminated samples were 826 and 196 μg toxin kg^−1^ maize for FB_1_ and FB_2_, respectively.

Multi-factor ANOVAs were carried out to study the impact of the agronomic factors on the response variables at harvest.

Neither FB_1_ nor FB_2_ concentrations in maize at harvest date were statistically significantly affected by any of the agronomic factors. DON content of the grains at harvest date was statistically significantly affected by the tillage system (see Table 2). Maize planted under IT had higher DON contamination (2695 μg DON kg^−1^ maize on average) than maize planted using DD (474 μg DON kg^−1^ maize on average).

It has been reported that residues of crops that were infected with *Fusarium* constitute an inoculum of the fungus for the following crop [19,20,21,22]. This inoculum tends to be particularly abundant in the case of maize [23]. Therefore, according to many authors, the removal, destruction or burial of infected crop residues is likely to reduce the *Fusarium* inoculum for the following crop, making IT a better choice than DD for controlling mycotoxin-producing fungal inoculums [19,21,22]. Mansfield, De Wolf and Kuldau (2005) reported that the DON concentration of ensiled maize was lower in maize planted using a moldboard till than in maize planted using no tillage [24]. Dill-Macky and Jones (2000) studied the DON contamination of wheat following corn, wheat or soybean, using different tillage systems [25]. DON levels were lower in wheat planted using moldboard ploughing following corn or wheat in comparison to wheat planted using no tillage following the same crops. No significant differences in DON levels in wheat were observed between the two tillage systems when the previous crop was soybean, as *F. graminearum* is not considered a pathogen of soybeans. Obst, Lepschy-Von Gleissenthall and Beck (1997) stated that the use of minimum tillage instead of mouldboard ploughing after a maize crop could result in a 10-fold increase in DON contamination of the following wheat crop [26]. Schöneberg et al. (2016) demonstrated that barley from fields with ploughed soils showed significantly less *F. graminearum* and DON content than barley from reduced tillage fields, regardless of the previous crop [27]. On the other hand, Roucou, Bergez, Méléard and Orlando (2022), who collected data from a total of 2032 maize fields located in France between 2004 and 2020, found that DON contamination in maize was not significantly different whether the crop residues of the previous year were adequately managed (mostly through soil tillage) or not [28]. Supronienė et al. (2012) studied the effect of different tillage practices (conventional tillage, reduced tillage and no tillage) on mycotoxin contamination in winter and spring wheat, but no clear relationship could be observed [29]. Furthermore, Kaukoranta, Hietaniemi, Rämö, Koivisto and Parikka (2019), who analyzed survey data from 804 spring-oat fields, found that the DON concentration of the oats was the same or higher under ploughing than under non-ploughing conditions [30]. Our results are closer to those of Kaukoranta et al. (2019), as we found a significantly higher DON contamination in maize planted under IT than in maize planted using DD.

Tillage operations can affect both the soil structure and the crop productivity [31]. Unlike no tillage, IT exposes soil to erosive agents such as wind and water. The impact of water drops induces the degradation of the soil by the breakdown of water-stable aggregates, causing soil crusting [32,33]. Soil crusting negatively affects seedling emergence, reduces water infiltration rates and water storage capacity, favors runoff, diminishes organic matter, and can cause overland flow [31,32,34]. Soils rich in silt and fine sand, such as the one in this study, are highly susceptible to soil crusting [35]. As we observed the presence of pools of water only in IT plots, most probably caused by soil crusting, we hypothesize that in these plots the pools of water created high air humidity conditions, favoring the growth of moulds throughout the whole cultivation period, including DON-producing moulds. In fact, it has been stated that under moist conditions the production of macroconidia, the production of ascopores and the ejection of ascospores of *F. graminearum* are favored [36,37,38]. That would help explain the higher DON contamination in maize planted under IT in comparison to maize planted using DD.

Another hypothesis that supports our results is that tillage affects soil fauna, which in turn can have an impact on *Fusarium* species. Earthworms are known for breaking down organic matter and promoting nutrient cycling along with soil microbiota, and for improving soil structure, soil porosity, soil water retention capacity, root distribution, plant growth and plant health. Frequent tillage adversely affects many earthworm species, especially those linked to the surface layers (epigeics and anecics) [39,40,41,42]. When the soil is turned over, earthworms are injured and killed, their burrows are broken, their food sources are buried, and they become exposed to harsh environmental conditions and predators. [39,40,41,43,44]. The common earthworm (*Lumbricus terrestris*) is one of the most important anecic earthworms and is capable of incorporating plant litter into the soil and decomposing it. Oldenburg, Kramer, Schrader and Weinert (2008) and Schrader, Kramer, Oldenburg and Weinert (2009) demonstrated that *L. terrestris* accelerates the degradation of *Fusarium* biomass and DON in the wheat straw layer, and that this earthworm is more attracted to highly *Fusarium*-infected and DON contaminated wheat straw than less infected and contaminated wheat straw [45,46]. *L. terrestris* is likely to prefer the contaminated straw as its N-content and digestibility are enhanced due to fungal colonization. Thus, *L. terrestris* most probably reduces *Fusarium* biomass in maize straw too, and consequently, minimizes *Fusarium* infection and DON contamination of maize cobs. Therefore, as the population of *L. terrestris* is smaller in IT plots, a lower DON contamination in maize planted under DD is expected than in maize planted under IT. In our case, we did not sample earthworms during the experiment. However, Santiveri Morata, Cantero-Martínez, Ojeda Domínguez and Angás Pueyo (2004) studied the population of earthworms in the same field where this study was performed, and found that under DD the population of worms was higher than in more aggressive tillage systems [47].

The moisture content of the grains at harvest date was statistically significantly affected by the crop diversification and the tillage system. Moisture was higher in SC maize (21.33% on average) than in LC maize (16.93% on average), and higher in maize under IT (20.45% on average) than in maize planted using DD (17.82% on average). Likewise, the a_w_ of the grains was significantly affected by the crop diversification, being greater in SC maize than in LC maize (0.927 and 0.897 on average, respectively). It should be noted, though, that the differences in moisture and a_w_ between different maturity groups could easily be modified by the harvesting dates. 

It has been reported that no tillage is associated with soil with a higher water holding capacity and higher soil moisture in surface soil layers in comparison with IT [48,49]. Therefore, one might think that the maize kernels obtained from DD-planted maize would have a higher moisture than maize kernels obtained from maize under IT, but that was not the case in our study. 

The log of total fungal contamination was significantly affected by the crop diversification (*p*-value = 0.046), being higher in LC maize (5.28 on average) than in SC maize (4.81 on average).

No effect of the agronomic factors was observed in the log of *Fusarium* spp., FB_1_ or FB_2_ contaminations. In this context, Ono et al. (2011) observed no significant differences in the *Fusarium* sp. counts and the fumonisin concentrations between non-tilled and conventional-tilled maize [50]. Similarly, Ariño et al. (2009) found no significant differences in the fumonisin contents of maize planted using minimum tillage and ploughing [51]. Even so, it is necessary to emphasize that the low incidence of FB_1_ and FB_2_ contamination on maize at harvest (12.5%) makes it rather difficult to observe differences in the concentration of these toxins due to agronomic factors.

Regarding how N fertilization can affect fumonisin contamination in maize, previous research has shown contrasting results. A shared vision is that a balanced fertilization is the best approach to minimize fumonisin concentrations, as stress due to N deficiency or high N rates can significantly raise fumonisin levels [50,52,53,54,55,56]. In our study, no differences in fumonisin contamination were observed between 0 N and High N fertilization rates. 

### 2.2. Correlations between the Studied Variables at the Harvest Date

**Figure 1:**
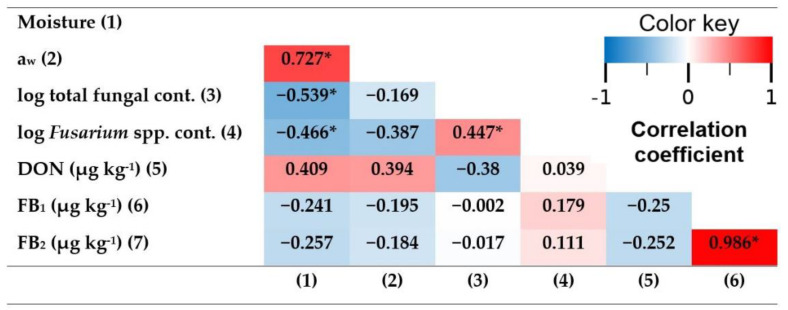
Correlation matrix heatmap based on the correlation coefficients from the PCA at harvest date. A darker blue color indicates a stronger negative correlation, while a darker red color indicates a stronger positive correlation. * indicates a significant correlation (*p*-value < 0.05).

Principal Component Analysis was performed in search of correlations between response variables at harvest (see correlation matrix heatmap in Figure 1). The variables studied were moisture, a_w_, the log of total fungal contamination, the log of *Fusarium* spp. contamination, and the different mycotoxin contaminations (DON, FB_1_ and FB_2_). Following the criteria of choosing the principal components with eigenvalues > 1, three principal components were taken, which accounted for 81.32% of the total variance. 

Few variables were significantly correlated. FB_1_ contamination was significantly positively correlated with FB_2_ contamination (r = 0.986, *p*-value < 0.001). That is in accordance with the results of Carbas et al. (2021) and Cao et al. (2013), who also found significant positive correlations between FB_1_ and FB_2_ contaminations (r = 0.96 and r = 0.99, respectively) [57,58].

There was a significant positive correlation between the log of total fungal contamination and the log of *Fusarium* spp. contamination, indicating that *Fusarium* spp. is of considerable relevance to total fungal contamination.

Moisture was significantly positively correlated with a_w_ (r = 0.727, *p*-value = 0.001), and significantly negatively correlated with the log of total fungal contamination (r = −0.539, *p*-value = 0.016) and with the log of *Fusarium* spp. contamination (r = −0.466, *p*-value = 0.035). Cao et al. (2013) also described a significantly negative correlation between moisture and *Fusarium* spp. contamination (r = −0.68, *p*-value < 0.05).

*Fusarium* spp. contamination at harvest date was not significantly correlated with the concentration of any of the studied mycotoxins (DON, FB_1_ and FB_2_) in the same period. This could be explained by there being non-DON/FB_1_/FB_2_-producing *Fusarium* spp. strains colonizing our maize, and/or because a higher count of DON/FB_1_/FB_2_-producing *Fusarium* spp. at harvest date does not necessarily imply a higher concentration of these mycotoxins. Factors such as a_w_, temperature and relative humidity can affect mycotoxin production [59,60]. Similarly, Lanza et al. (2017) found no association either between fumonisin levels and the frequency of *Fusarium* spp. in maize kernels [61]. On the other hand, Schöneberg et al. (2016) found that *F. graminearum* was positively correlated with DON content in barley (r = 0.72, *p*-value < 0.001) [27].

No significant correlations were observed between DON and FB_1_ or FB_2_ concentrations. That is consistent with the bibliography, as it has been described that in maize DON is produced primarily by *F. graminearum* and *F. culmorum*, while FB_1_ and FB_2_ are mainly produced by *F. verticillioides*, *F. proliferatum* and *F. subglutinans* [4,5,6,7].

DON was positively correlated with moisture and a_w_, but the correlations were not significant (*p*-values of 0.058 and 0.066, respectively).

### 2.3. Effect of Time and Temperature on Maize Moisture, a_w_, Microbial Counts and Mycotoxin Contamination after Harvest

Multi-factor ANOVAs were carried out to determine the effect of time and temperature (15 or 25 °C) on the studied variables. On one side, moisture, a_w_ and microbial counts were studied on days 0, 4, 7 and 10. On the other side, mycotoxin contamination was studied on days 0, 5 and 10. All the data are available in a spreadsheet in the Supplementary Materials.

**Figure 2:**
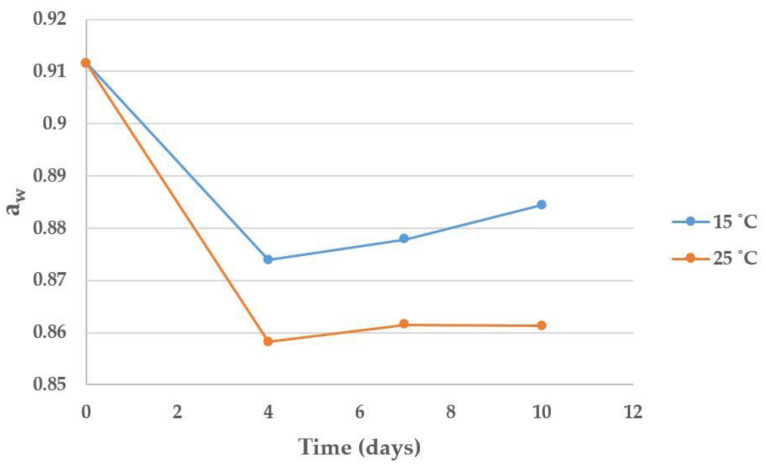
Influence of time and temperature on the evolution of a_w_.

No significant effect of time nor temperature was observed on the moisture, the total fungal contamination or the *Fusarium* spp. contamination during the 10 days of the experiment. By contrast, the variable time significantly affected the evolution of a_w_ (*p*-value = 0.001), which dropped on day 4 for both temperatures (Figure 2). Statistically significant differences were observed between a_w_ on day 0 and a_w_ on days 4, 7 and 10.

DON, FB_1_ and FB_2_ contaminations were not affected by time or temperature, although in the case of DON time was close to being significant (*p*-value = 0.078). Thus, statistically significant differences were observed in DON concentrations between days 0 and 5 (*p*-value = 0.049) but not between days 0 and 10 (*p*-value = 0.051) or days 5 and 10 (*p*-value = 0.989) (see Table 3). Regarding FB_1_ and FB_2_ contamination, the tendency was the same as that at harvest: a low prevalence of these toxins. On days 5 and 10, only 15.63 and 18.75% of samples contained at least one of the studied fumonisins. The average contamination of contaminated samples on days 5 and 10 was 1938 and 1709 μg toxin kg^−1^ maize for FB_1_, and 1068 and 1279 μg toxin kg^−1^ maize for FB_2_.

As an increase in DON concentration was observed in the 0–5 days period, and *Fusarium* spp. counts remained stable during the whole 10 days period, the absence of DON production during the 5–10 days period could be attributed to the drop in a_w_ during the first 4 days. If a_w_ levels had remained constant since harvest, DON contamination most likely would have increased continuously. Considering these results, we could say that under the tested temperatures (15 and 25 °C), there are DON-producing *Fusarium* spp. species in maize that can produce DON at an approximate a_w_ of at least 0.91, while at an a_w_ of 0.88 they can no longer produce this toxin. 

Our results are in line with those obtained by Comerio, Fernández Pinto and Vaamonde (1999), who studied the DON production of *F. graminearum* in wheat at different a_w_ [62]. They found that at an a_w_ = 0.925 DON was produced, but not at a_w_ = 0.900; therefore, the limiting a_w_ for DON production under those conditions was close to 0.900. Other studies have suggested slightly higher values under similar conditions. Ramirez, Chulze and Magan (2006) studied the DON production of *F. graminearum* on wheat and found mycotoxin production at a_w_ = 0.95 at the temperatures of 15, 25 and 30 °C, but they did not find DON production at a_w_ = 0.93 under any temperature [63]. Schmidt-Heydt, Parra, Geisen and Magan (2011) found that *F. culmorum* and *F. graminearum* could produce DON at an a_w_ = 0.93 at 25 °C in YES medium after a 9 day incubation, but not at a_w_ = 0.90 under any of the tested conditions [64].

## 3. Conclusions

At harvest, all maize samples were contaminated with DON (1584 ± 1578 μg DON kg^−1^ maize), while only 12.5% of the maize samples were contaminated with FB_1_ and FB_2_ (average contaminations of contaminated samples were 826 and 196 μg toxin kg^−1^ maize, respectively). No effect of the crop diversification or the N fertilization rate was observed on the maize DON contamination. The only agronomic factor that significantly affected the DON content of grains was the tillage system. Maize planted under IT presented a greater DON contamination (2695 μg DON kg^−1^ maize on average) than maize planted using DD (474 μg DON kg^−1^ maize on average). Two main reasons support these results. The first reason is that in IT plots the degradation of the soil resulting from the continuous tillage caused soil crusting, which induced the formation of pools of water, creating high air humidity conditions, which favored the growth of DON-producing moulds. The second reason is that the frequent tillage in IT plots causes a decrease in the population of *L. terrestris*. This earthworm is likely to reduce *Fusarium* infection and DON contamination in maize straw. Consequently, maize cobs under DD are expected to be less infected and contaminated. Hence, DD would be a better approach than IT not only in terms of controlling DON contamination, but also from the agronomic point of view. More studies that employ long-term IT and DD plots are needed to assess precisely how the tillage system can influence the mycotoxin contamination of grains.

No significant correlations were found between the log of *Fusarium* spp. contamination at harvest date and the concentration of any of the studied mycotoxins in the same period. 

During the 10-day storage, no effect of time or temperature was observed on the moisture, the total fungal contamination, the *Fusarium* spp. contamination or the FB_1_ and FB_2_ contaminations. Time affected the evolution of a_w_, which fell on day 4 for both temperatures. DON concentration on day 5 was significantly higher than on day 0, but there were no significant differences between days 5 and 10. Therefore, it is predictable that continued DON production was held back by the a_w_ drop in the first 4 days of storage, meaning the minimum a_w_ for the DON-producing species colonizing our maize to produce this toxin is around 0.91.

To our knowledge, this is the first study that relates soil crusting and the consequent formation of pools of water in maize plots under IT with a higher DON grain contamination in comparison with maize plots under DD, and is also the first work to question how the harvest-till-drying period of maize can affect fungal and mycotoxin contamination.

## 4. Materials and Methods

### 4.1. Climate and Soil Characteristics, Experimental Design and Crop Management

Maize was planted in an experimental field in Agramunt, NE Spain (41°48′ N, 1°07′ E, 330 m asl). The soil in this area is classified as xerofluvent typic [65]. Many soil characteristics were measured: the average pH of the soils was (H_2_O, 1:2.5) 8.5; the electrical conductivity (1:5) was 0.15 dSm^−1^; the soil organic carbon (SOC) concentration (0–30 cm) was 8.6 g kg^−1^; the water available holding capacity (between −33 kPa and −1500 kPa) was 10% (*v*/*v*). The climate of the area is semiarid Mediterranean with a continental trend. Climate was monitored with a weather station placed in the experimental field. During the last 30 years, the mean annual precipitation was 442 mm, the mean annual temperature was 14.6 °C, and the mean annual potential evapotranspiration (PET) was 855 mm. The winter is cold, with some days below 0 °C in January. For that, soil temperature does not reach 8 °C until the beginning of April, when the planting date for maize starts. Additionally, the climate imposes hot summers, reaching temperatures over 35 °C in July and August. 

The experimental design was a split-plot with 3 blocks. The plots were 50 m × 3 m = 150 m^2^ and 4 rows of maize were planted in each plot (rows spaced 73 cm apart). Three agronomic factors were evaluated: the crop diversification, the tillage system and the N fertilization rate. For the crop diversification, a monocropping long-cycle maize (LC maize) (FAO 700 maturity group, Pioneer’s P1570 hybrid) was compared against a legume–maize double cropping, using short-cycle maize (SC maize) (FAO 400 maturity group, Pioneer’s P0312 hybrid) as the main crop and vetch (*Vicia sativa* L., var. Prontivesa) as the secondary crop.

**Figure 3:**
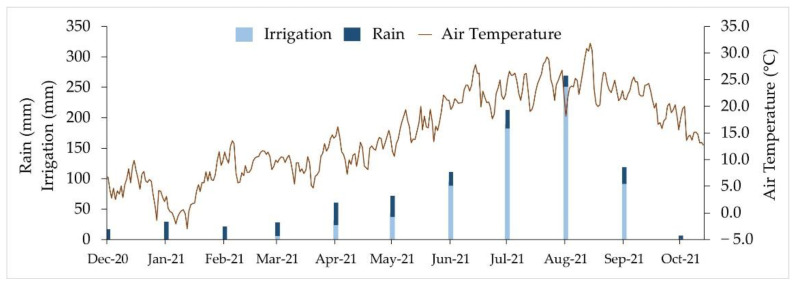
Irrigation and meteorological conditions in the experimental field.

In the case of the tillage system, intensive tillage (IT) and direct drilling (DD) were studied. IT consisted of subsolate (35 cm depth), disc harrow and rototiller, while DD consisted of the application of herbicide (1.5 L ha^−1^ of 36% glyphosate [*N*-(phosphonomethyl)-glycine]) and sowing directly the seeds into the soil. In reference to the N fertilization rate, a zero N rate (0 N) and high N rate (High N) were evaluated. The rate of mineral fertilization applied was 400 kg N ha^−1^ for LC maize, while it was reduced to 300 kg N ha^−1^ in SC maize because of the possible fixation of the preceding legume crop. N fertilization was distributed between 2 top-dressing fertilizations with ammonium nitrate (34.5% N), with a rate of 150 kg N ha^−1^ in each one at stages V3–V5 (May in LC maize and June in SC maize) and V7–V8 respectively (June in LC maize and July in SC maize). In addition, for LC maize, a 100 kg N ha^−1^ pre-emergence fertilization was carried out during April with urea (46% N). The experiment was carried out over 3 years (2019, 2020 and 2021), although the present study was carried out with the third year’s harvest. LC and SC maize were seeded in April and June, respectively. Accordingly, its flowering took place in July and August, respectively. Vetch was sown in December. In both maturity groups, the planting rate was 90,000 seeds ha^−1^, with a row spacing of 73 cm. In the case of vetch, the planting density was 267 plants m^−2^. All maize plots received equally a pre-emergence herbicide treatment with 7 L ha^−1^ of Primextra Gold (Terbuthylazine 18.75% + *S*-Metolachlor 31.25% (SE) *w*/*v*). For each tillage system and plant species, the harvest residue was treated differently. In the case of maize and IT, it was integrated into the soil by tillage, whereas in DD, it was chopped and spread on the soil surface. Vetch was harvested for forage at a cutting height of 5 cm, so all the biomass was exported from the plots. The irrigation rate was determined using Dastane’s methods [66] for calculating crop water requirements on a weekly basis. Irrigation was carried out by sprinkling, starting in March and ending in October. The amount of irrigation used and mean meteorological conditions in the experimental field, obtained from an on-site weather station, are shown in Figure 3.

### 4.2. Maize Harvesting and Storage

Cob samples of a total of 16 different plots were taken (2 cultivars × 2 tillage systems × 2 N fertilization rates × 2 blocks). Both cultivars were harvested when the maize was close to the commercial moisture (18 %). That was the 21st and 26th of October 2021 for LC and SC maize, respectively. On the harvest day, around 1.5 kg of maize (approximately 8–10 maize cobs) was sampled from each plot. The different maize cobs were collected throughout the entire plot, being representative of the area of study. As not to alter the microbiota of the samples, the cobs were picked up using different sterile nitrile gloves for each plot. The maize from each plot was deposited and transported in a different sterile plastic bag. In the laboratory, cobs were shelled under sterile conditions in a laminar flow cabinet. The kernels from each plot were split into two different sterile plastic bags, which were kept at different temperatures: 15 or 25 °C, for 10 days. Those specific temperatures were chosen to simulate the average maximum and minimum daily temperatures in the area at the time of harvest. 

### 4.3. Laboratory Determinations

Different determinations were performed on the harvest day (day 0) and the following days for the maize from each plot. Moisture (%), water activity (a_w_), total fungal contamination (CFU g^−1^ maize) and *Fusarium* spp. contamination (CFU g^−1^ maize) were determined on days 0, 4, 7 and 10. DON, FB_1_ and FB_2_ contamination were determined on days 0, 5 and 10.

#### 4.3.1. Moisture

Approximately 15 g of maize kernels were precisely weighed into pre-weighed glass jars. The jars were put in an oven (JP Selecta 210, JP Selecta S.A., Abrera, Spain) at 105 °C for 16 h, and after that period were weighed again. The moisture was calculated according to Equation (1). Three replicates were carried out for each plot, storage time and storage temperature. Average moisture and standard deviation were calculated.

(1) $$Moisture\;\left(\%\right)=\frac{W_{0}-W_{f}}{W_{0}-W_{j}}*100$$

where $W_{0}$ is the weight of the glass jar and the maize before drying, $W_{f}$ is the weight of the glass jar and the maize after drying, and $W_{j}$ is the weight of the glass jar.

#### 4.3.2. Water Activity (a_w_)

The a_w_ of whole maize kernels for each plot, storage time and storage temperature was measured using the AquaLab Series 3 TE (AquaLab S.L., Sabadell, Spain). A sample of about 3 g was introduced into the water activity meter, and a_w_ was properly read.

#### 4.3.3. Total Fungal Contamination and *Fusarium* spp. Contamination

One maize sample from each plot, storage time and storage temperature was analyzed for total fungal contamination and *Fusarium* spp. contamination. Approximately 20 g of kernels was ground using a disinfected IKA A11 (IKA^®^-Werke GmbH & Co. KG, Staufen, Germany) mill for 30 s. Ten grams of the resulting flour was weighed in a sterile Stomacher bag with a lateral filter. Then, 90 mL of sterile saline peptone water was added to the bag (10^−1^ dilution). The flour and the saline peptone water were mixed in a laboratory blender (Stomacher 400, Seward Ltd., Worthing, UK) for 120 s at normal speed. A series of dilutions were prepared based on the filtered extract using saline peptone water (up to the 10^−6^ dilution). Then, 0.1 mL of each dilution was plated into Petri plates containing Chloramphenicol Glucose Agar (CGA) (for total fungal contamination) or Malachite Green Agar 2.5 (MGA) (a selective medium for *Fusarium* spp.). 

The inoculum was spread across the Petri plates with a Digralsky spreader, and the plates were incubated upside down at 25 °C. Plate readings were performed after 3 days of incubation for CGA plates and 4 days of incubation for MGA plates. 

#### 4.3.4. DON, FB_1_ and FB_2_ Contamination

##### Extraction of DON, FB_1_ and FB_2_

One sample from each plot, storage time and storage temperature was analyzed for its DON, FB_1_ and FB_2_ content. An amount of 17 g of each sample were ground in a IKA A11 mill for 30 s. Seven grams of ground maize were transferred into a 50 mL Falcon tube for DON analysis, and another 7 g of ground maize were put into another 50 mL Falcon tube for FB_1_ and FB_2_ analysis.

##### DON Extraction and Sample Preparation

DON extraction and analysis were based on the study of Borràs-Vallverdú, Ramos, Marín, Sanchis and Rodríguez-Bencomo (2020) [67]. An amount of 1.4 g of NaCl and 40 mL of Milli-Q water were added to the Falcon tube with the ground maize. The mixture was vortexed for 30 s and ultrasound-treated with the Bransonic M2800H-E (Branson Ultrasonic SA, Carouge, Switzerland) at maximum power for 15 min. After that, the Falcon tubes were centrifuged in a Hettich 320R centrifuge (Andreas Hettich GmbH & Co. KG, Tuttlingen, Germany) at 8965× *g* for 10 min at 20 °C. The supernatant was vacuum filtered using 90 mm glass microfiber filters (Whatman, Buckinghamshire, UK). DonPrep immunoaffinity columns (Biopharm AG, Darmstadt, Germany) were prepared by adding 10 mL of Milli-Q water. Then, 8 mL of the filtered supernatant was collected and passed through the immunoaffinity column. After that, 1.5 mL of methanol was added to elute the toxin. Backflushing was done three times, and then another 0.5 mL of methanol was passed through the column. The 2 mL of collected methanolic extract was evaporated at 40 °C (Stuart SBH200D/3 block heater, Cole-Parmer©, Staffordshire, UK) under a gentle stream of N_2_. The residue was re-suspended in 0.8 mL of MeOH:H_2_O 10:90 (*v:v*), vortexed, filtered through 0.22 µm PTFE filters and analyzed by HPLC-DAD according to the following section.

##### DON HPLC-DAD Analysis

HPLC-DAD determination of DON was performed using an Agilent Technologies 1260 Infinity HPLC system (Santa Clara, CA, USA) coupled with an Agilent 1260 Infinity II Diode Array Detector (DAD). A Phenomenex^®^ Gemini C18 column (Torrance, CA, USA) was used (150 × 4.6 mm, 5 µm particle size, 110 Å pore size). Absorbance reading was performed at 220 nm. Three mobile phases were prepared: phase A (methanol:water 10:90, *v:v*), phase B (acetonitrile:water 20:80, *v:v*) and phase C (100% methanol). The gradient applied was as follows: 0 min 100% A; 10 min 60% A and 40% B; 13 min 60% A and 40% B; 15 min 100% C; 25 min 100% C; 29 min 100% A until 40 min (for re-equilibrating the column). The flow rate was set at 1 mL/min. The column temperature was 40 °C, and the injection volume was 50 µL. DON retention time was 10.0 min. Quantification was carried out by using DON calibration curves prepared in methanol:water 10:90, *v:v*.

LOD and LOQ, considered as three and ten times the signal of the blank, respectively, were 12.6 and 42.0 μg kg^−1^. Recovery was calculated using artificially DON-contaminated maize, extracting and analysing the mycotoxins as previously stated. Recovery was studied per triplicate at three different DON concentrations: 2.286, 1.143 and 0.571 μg DON kg^−1^ maize. The respective average recoveries and standard deviations were 81.7 ± 9.5, 87.4 ± 13.3 and 91.3 ± 14.5%.

##### FB_1_ and FB_2_ Extraction and Sample Preparation

Fumonisin extraction and analysis were based on the study of Belajova and Rauova (2010) [68]. An amount of 1.4 g of NaCl and 35 mL of H_2_O:ACN:MeOH 50:25:25 (*v:v:v*) were added to the Falcon tube with the ground maize. The mixture was vortexed for 30 s and ultrasound-treated with the Bransonic M2800H-E (Branson Ultrasonic SA, Carouge, Switzerland) at maximum power for 15 min. After that, the Falcon tubes were centrifuged in a Hettich 320R centrifuge (Andreas Hettich GmbH & Co. KG, Tuttlingen, Germany) at 8965× *g* for 10 min at 20 °C. The supernatant was vacuum filtered using 90 mm glass microfiber filters (Whatman, Buckinghamshire, UK). The solution to be analyzed was prepared by mixing 3.5 mL of the filtered supernatant with 46.5 mL of PBS in another 50 mL Falcon tube. The whole content of the Falcon tube was passed through a Fumoniprep immunoaffinity column (Biopharm AG, Darmstadt, Germany). After that, 1.5 mL of methanol was added to collect the toxin. Backflushing was done three times, and then 1.5 mL of Milli-Q water was passed through the column. The 3 mL of collected solution was evaporated at 40 °C (Stuart SBH200D/3 block heater, Cole-Parmer©, Staffordshire, UK) under a gentle stream of N_2_. The residue was re-suspended in 0.8 mL of MeOH:H_2_O 50:50 (*v:v*), vortexed, filtered through 0.22 µm PTFE filters and analyzed by HPLC-FLD according to the following section.

##### FB_1_ and FB_2_ HPLC-FLD Analysis

HPLC-FLD determination of FB_1_ and FB_2_ was performed using an Agilent Technologies 1260 Infinity HPLC system (Santa Clara, CA, USA) coupled with an Agilent 1260 Infinity Fluorescence Detector (FLD). A Phenomenex^®^ Kinetex PFP column (Torrance, CA, USA) was used (150 × 4.6 mm, 5 µm particle size, 110 Å pore size). Excitation and emission were performed at 335 and 460 nm, respectively. Three mobile phases were prepared: phase A (acetonitrile), phase B (methanol) and phase C (0.1% acetic acid). The gradient applied was as follows: 0 min 15% A and 85% C; 10 min 5% A, 61% B and 34% C; 14 min 5% A, 61% B and 34% C; 16 min 5% A, 72% B and 23% C; 20 min 15% A and 85% C (for re-equilibrating the column). The flow rate was set at 1.2 mL/min. The column temperature was 40 °C, and the injection volume was 50 µL. FB_1_ and FB_2_ retention times were 15 and 17.8 min, respectively. Quantification was carried out by using FB_1_ and FB_2_ calibration curves prepared in methanol:water 50:50, *v:v*.

Prior to injection, samples were derivatized. The derivatization mixture (DM) for the analysis of fumonisins was prepared as follows: 40 mg of ortho-phthaldialdehyde was dissolved in 1 mL of methanol and diluted in 10 mL of 0.1 M disodium tetraborate. Then, 50 μL of 2-mercaptoethanol was added and the mixture was vortexed. The prepared mixture was stored in an amber glass vial at 4 °C for a maximum of 7 days. The injector was programmed to draw 37.5 µL of DM and 12.5 µL of the sample to be analyzed, and then we mixed them for 0.3 min before injection.

LOD and LOQ, considered as three and ten times the signal of the blank, were 10.0 and 33.3 μg kg^−1^ for FB_1_ and 16.0 and 53.3 μg kg^−1^ for FB_2_, respectively. Recovery was calculated using artificially fumonisin-contaminated maize, extracting and analysing the mycotoxins as previously stated. Recovery was studied per triplicate at three different fumonisin concentrations: 0.855 + 0.855, 0.57 + 0.57 and 0.285 + 0.285 (μg FB_1_ + μg FB_2_) kg^−1^ maize. For FB_1_, the respective average recoveries and standard deviations were 82.1 ± 8.7, 86.8 ± 9.2 and 77.0 ± 9.5%. For FB_2_, those values were 102.6 ± 21.5, 101.6 ± 27.7 and 88.6 ± 27.6%.

### 4.4. Reagents and Chemicals

DON was from Romer Labs (Tulln, Austria). FB_1_ and FB_2_ were from Sigma (St. Louis, MO, USA), ortho-phthaldialdehyde was from Merck (Darmstadt, Germany) and 2-mercaptoethanol was from Scharlau (Sentmenat, Spain). Methanol HPLC grade, acetonitrile HPLC gradient grade and NaCl were from Fisher Scientific UK Limited (Loughborough, UK). 

CGA was from Biokar (Barcelona, Spain). MGA was prepared in the laboratory according to Castellá et al. (1997) [69]. Peptone was from Biokar (Barcelona, Spain), KH_2_PO_4_ and chloramphenicol were from Scharlau (Sentmenat, Spain) and MgSO_4_·7 H_2_O was from Quality chemicals (Esparreguera, Spain). Malachite green (C_48_H_50_N_4_O_4_·2C_2_H_2_O_4_) was from Probus (Badalona, Spain) and agar was from Condalab (Torrejón de Ardoz, Spain).

### 4.5. Statistics

Statistical analyses were carried out using the SPSS program for Windows (version 22) (IBM Corp., Armonk, New York, NY, USA; https://www.ibm.com/es-es/analytics/spss-statistics-software, access on 28 August 2022). The significance level was established at 0.05. Descriptive statistics, Principal Compounds Analysis and multiple-factor ANOVAs were performed. LSD tests were used to evaluate significantly statistical differences among groups in a variable. Graphics were drawn using Microsoft Excel 2013.

## Figures and Tables

**Table 1:** DON contamination in maize at harvest.

FAO Maturity Group/Cropping System	Fertilization	Tillage System	Average DON Contamination (μg Toxin kg^−1^ Maize)
400/SC	0 N	DD	440
		IT	2848
	High N	DD	566
		IT	4406
700/LC	0 N	DD	654
		IT	791
	High N	DD	236
		IT	2734

SC: short cycle; LC: long cycle; 0 N: zero nitrogen rate; High N: high nitrogen rate; DD: direct drilling; IT: intensive tillage.

**Table 2:** Test of between-subjects effects for DON contamination at harvest date.

	SS	df	MS	F	Sig.
Crop diversification	3.697	1	3.697	1.292	0.289
N. fert. rate	2.574	1	2.574	0.900	0.371
Tillage system	19.729	1	19.729	6.897	**0.030**
Crop diversification × N fert. rate	0.006	1	0.006	0.002	0.964
Crop diversification × Tillage system	3.260	1	3.260	1.140	0.317
N fert. Rate × Tillage system	3.598	1	3.598	1.258	0.295
Crop diversification × N fert. Rate × Tillage system	0.216	1	0.216	0.075	0.791

R Squared = 0.591 (Adjusted R Squared = 0.233). SS: sum of squares; df: degrees of freedom; MS: Mean Square; F: F-value; Sig.: significance value. Bold value is the only statistically significant factor.

**Table 3:** Influence of time and temperature on the evolution of DON concentrations (μg DON kg^−1^ maize).

Temperature	DON Concentration (μg DON kg^−1^ Maize)		
	Day 0	Day 5	Day 10
15 °C	1584 ± 1932	2367 ± 2983	2649 ± 2349
25 °C	1584 ± 1932	3771 ± 3597	3469 ± 4300

Presented values correspond to mean and standard deviation.

## Data Availability

The data presented in this study are available in this article and Supplementary Materials.

## Supplementary Materials

The following supporting information can be downloaded at: https://www.mdpi.com/article/10.3390/toxins14090620/s1. Supplementary Document: Effect of Time and Temperature on Maize Moisture, aw, Microbial Counts and Mycotoxin Contamination after Harvest.
